# Effects of continuous cropping on bacterial community diversity and soil metabolites in soybean roots

**DOI:** 10.3389/fmicb.2025.1534809

**Published:** 2025-02-10

**Authors:** Liwei An, Xinnan Lu, Pengyu Zhang, Jiayao Sun, Baiming Cong, Rula Sa, Dexin He

**Affiliations:** ^1^Pratacultural College, Inner Mongolia Minzu University, Tongliao, China; ^2^Agriculturalc College, Inner Mongolia Minzu University, Tongliao, China; ^3^Inner Mongolia Agronomy and Animal Husbandry Technology Extension Center, Hohhot, China; ^4^Tongliao Institute of Agricultural and Animal Husbandry Sciences, Tongliao, China

**Keywords:** continuous cropping barrier, microbiome, soil metabolite, crop rotation, soybean

## Abstract

The alternating planting of corn and soybeans is regarded as an effective strategy in addressing the challenges faced in soybean cultivation. However, the precise mechanisms that control the bacterial microbiome in soybean roots in the soil, particularly in continuous cropping and rotational corn–soybean farming rotations, are remain unclear. This study employed both field and pot positioning experiments, using high-throughput and generic metabolomics sequencing techniques to explore the dynamics between soybean plants, root microflora, and soil metabolites, especially in the context of continuous cropping and fluctuating corn–soybean crop rotation. The process that included rotating corn soybeans significantly enhanced their grain yield, dry weight, soil nitrogen concentration, urease activity, as well as the accumulation of nitrogen, phosphorus, and potassium in various plant organs, compared to the traditional practice of continuous soybean cultivation. There is a significant reduction in the transit of bacterial operational taxonomic units (OTUs) from the rhizosphere to the endosphere through rhizoplane. The number of bacterial OTUs that are consumed and enriched on rhizoplane is greater than those that are enriched and absorbed in the endosphere. Continuous cropping practices significantly boost Burkholderiales, whereas chloroplast microorganisms significantly improve crop rotation techniques. Soil environmental factors, such as urease and accessible phosphorus, are crucial in establishing the relative prevalence of Rhodanobacter and other bacterial groups. Soil metabolites, such as benzyl alcohol, show a positive correlation with Cyanobacteria, while acidic compounds, such as D-arabinitol, are positively linked with Burkholderiales. This study indicates that the rotation of corn and soybean crops facilitates the growth of soybeans, increases nutrient accumulation in both plants and soil, enhances the presence of beneficial bacteria, and improves soybean yields.

## 1 Introduction

A wide range of soil bacteria plays a critical role in the soil ecosystem. The number and diversity of soil bacteria serve as crucial baseline indicators for assessing soil quality (Sharma et al., 2011). Various research studies indicate that alternating crops and intercropping enhance soil bacterial diversity and abundance to different extents in contrast to regular cropping techniques. Following potato rotation and corn intercropping, there is a notable rise in soil bacterial diversity (Lu et al., 2023); similarly, cornflower intercropping leads to a noticeable increase in peanut soil bacterial levels, with Alternaria fungi being evidently less than in continuous cropping, and Sphingomonas considerably more than in continuous cropping peanuts (Qingkai, 2020); the bacterial population in corn–soybean rotation soil is significantly greater than in soybean continuous cropping. Proteobacteria, as the primary bacterial species, dominate soybean rotation soil, while Acinetobacter prevails in continuous soybean cropping soil (Pérez-Brandán et al., 2014). A plethora of research indicates considerable variations in soil microbial diversity across various ecosystem types, potentially due to shifts both in biotic and abiotic factors of the soil (Green et al., 2008). The research technology of soil bacterial communities has been advanced significantly with the development of society. Previous studies have mainly used techniques, such as plate counting and polymerase chain reaction denaturing gradient gel electrophoresis (PCR-DGGE) to investigate the number and community composition of bacteria in continuous soybean cropping (Tan et al., 2012). These technologies can be used to study the composition of conventional bacterial communities; however, from the perspective of temporal considerations and research depth, these technologies still exhibit identifiable limitations. At present, the emergence of the Illumina MiSeq high-throughput sequencing system provides us with many convenient conditions for further studying the composition and diversity of bacterial communities in continuous soybeans cropping.

Kang et al. conducted research on potatoes and discovered that rotating fava beans, unlike regular potato cropping, decreases ester levels in root secretions and boosts hydrocarbons, ketones, carboxylic acids, and amines (Yichen et al., 2020). Zhao et al. examined the substantial impact of grass growth on the metabolites in the soil of peach orchards (Baixia et al., 2024). Following the expansion of grass, there was a notable increase in the diversity of soil metabolites, with 13 metabolites exhibiting significantly reduced expression levels. This observation indicates an enhancement in tryptophan metabolism, fatty acid production, and the degradation of aromatic compounds. The varied metabolites that are notably abundant in tryptophan metabolism and pathways involving fatty acids are generally not directly linked to plant growth. Metabolites in pathways breaking down aromatic compounds such as phthalic acid, which are abundant in these, show a marked decrease during treatment with grass. Research indicates that phthalic acid commonly reacts with phenolic acids and is formed post-decomposition of corn stover, adversely influencing crop growth and seed sprouting (Liang et al., 2009). Lu et al. employed gas chromatography–mass spectrometry (GC–MS) to observe notable variations in the levels of metabolites such as alkanes, organic acids, and benzenes in rhizosphere soil, particularly between rice fallow and rice rapeseed rotation methods (Lu et al., 2019). Following extended periods of consistent cropping, the extensive application of fertilizer led to a rise in organic acidity, gradually making the soil acidic annually. This not only devastated the soil but also suppressed microbes that thrive in it, resulting in self-toxic effects within the soil (Chen et al., 2020). This study combines 16S rDNA sequencing and untargeted metabolomics to examine the structural composition of soybean root bacterial microbiota and the response of soil metabolites to continuous cropping. It revealed the critical role of soil microorganisms and metabolites in regulating the ecological functions of soybean continuous cropping soil. This study will enhance the understanding of the obstacles to continuous soybean cropping regarding changes in soil microorganisms and their metabolism.

## 2 Materials and methods

### 2.1 Crafting experiments and gathering samples

In 2015, the Liaozhong experimental facility of Shenyang Agricultural University (situated at 41°52′N, 122 °72′W, with an altitude of 5.5–23.5 m) conducted a positioning study using a continuous soybean cropping method, alternating planting of corn and soybean. This area’s climatic conditions fall under the semi-moist continental category of the southern temperate zone, characterized by an average rainfall of 640 mm, an annual average temperature of 8°C, and a mean yearly sunshine duration of 2,527 h. Essential soil nutrients include 1.26 g·kg^−1^ of total nitrogen, 11.15 g·kg^−1^ of organic matter, 37.30 mg·kg^−1^ of accessible phosphorus, and 161.78 mg·kg^−1^ of accessible potassium. This experiment will be sampled and measured in 2021 and 2022. Adopting a split zone design, different planting systems were used to treat [continuous cropping (LC, no fertilization), crop rotation (LR, no fertilization)] as the main zone, and fertilization treatment as the secondary zone (pure nitrogen: 90 kg·hm^−2^; pure phosphorus: 90 kg·hm^−2^; pure potassium: 90 kg·hm^−2^). The tested varieties are Liaodou 14 (soybean) and Dongdan 6531 (corn). Soybean field, repeated 3 times per plot, with 6 rows per plot, a total length of 15 m, an area of 54 m^2^ per plot, and a planting density of 150,000 plants per hectare. Select seeds with care prior to sowing, with 4 seeds per hole and a sowing depth of 3 cm. Interplanting should be carried out during the seedling stage, with 2 plants left in each hole; corn fields, repeated 3 times per plot, with 6 rows per plot, a total length of 15 m, an area of 54 m^2^ per plot, and a planting density of 50,000 plants per hectare. Two seeds per hole, sowing depth of 3–5 cm, interseedling stage, leaving 1 seedling per hole, conventional field management.

During 2021, soil samples were gathered during the soybean blooming (R1) and the grain replenishment (R6) phases. Three plots exhibiting consistent soybean plant growth were chosen randomly as biological duplicates for the soybean continuous cropping and corn–soybean rotation planting zones. Choose the top layer of topsoil, still unplanted (0–20 cm), between two ridges for an analysis of the soil’s physicochemical properties; randomly pick up five soybean plants from three evenly grown soybean crop plots and corn–soybean rotation, store them in sterile, self-sealing bags, swiftly put them in a dry ice incubator, and then return them to the lab, refrigerating at −80°C for future processing.

### 2.2 Processing of soil samples

Soil that forms part of the endosphere within 1–5-mm distance is known as rhizosphere soil (Beckers et al., 2017). This experiment’s technique to gather rhizosphere, root topsoil, and roots adhered to earlier established sampling protocols (Wang et al., 2012; Xiaodong et al., 2019). The procedure proceeds as such:

Gently touch the soybean roots for the natural shedding of large soil chunks. Employ a germ-free brush to pick up the soil (0–5 mm) clinging to the rhizosphere as rhizosphere soil, gather it aseptically, transfer it into a sterilization tube, and refrigerate it at −80°C to sample rhizosphere microbes and the soil metabolite.

Remove the rhizosphere soil from the examined plant using sterilized scissors, transfer its root system into a beaker with 100 mL of sterile water, agitate it for 30 min, extract the root system, and then dissolve the soil in the water to form the rhizoplane. Employ sterilized filter paper for absorption, filtration, collection, transfer into a sterilization tube, and preserve at −80°C for examining rhizoplane soil microbes.

Choose the soil’s root structure on rhizoplane, store it in a sterile self-sealing pouch, and keep it at −80°C as a specimen to analyze the microorganisms present in the endosphere.

### 2.3 Identification of physical and chemical elements in soil

Gu et al. assessed the soil acidity levels, nitrate and ammonium nitrogen, available phosphorus, and total phosphorus in both soil and potassium, among other factors (Xueyuan and Aiverson, 2020).

### 2.4 Soil enzyme activity determination

Soil urease (URE) is examined using the indigo phenol blue colorimetric technique (Xiao et al., 2008). The activity of soil N-acetyl-β-glucosidase (NAG) is evaluated through microplate fluorescent detection methods; meanwhile, leucine aminopeptidase (LAP) activity is measured using this technique (Saiya-Cork et al., 2002).

### 2.5 Dry matter weight

Choose three plants that grow evenly in R1 (flowering stage), R6 (filling period), and R8 (maturity period), dry them at 105°C and dehydrate them at 85°C, then assess and compute the dry matter weight of every organ.

### 2.6 Yield

Throughout the R8 crop phase of soybeans, three lines of 3 m soybeans were harvested from different plots to determine their yield, measured at 13% moisture level and transformed into per-hectare yield.

### 2.7 Nutrient accumulation and distribution

Crush the dry matter samples taken from R1, R6, and R8. Employ the H_2_SO_4_-H_2_O_2_ technique for sample digestion and preparing the test solution for subsequent use. The Kjeldahl nitrogen analyzer (KJELTECTM8400), functioning autonomously, was used to assess the nitrogen, phosphorus, and potassium levels in stem, leaf, petiole, pod, grain, and entire plants.

### 2.8 Soil 16S rDNA sequencing

Forwarded the experimental sample to Wuhan Maiweier Biotechnology Co., Ltd. for the extraction and further examination of total DNA from the soil. Employing the CATB technique for isolating soil samples is followed by using agarose gel electrophoresis to determine the DNA’s concentration and purity post-extraction. Use sterile water to reduce the sample’s concentration to 1 ng/μL, then dilute it to extract a DNA template for PCR amplification. We used agarose gel and Qiagen’s adhesive recovery kit to isolate the product. Additionally, PCR amplification focused on 16S ribosomal RNA (rRNA), utilizing specific primers of bacteria by 515F (GTGCCAGCMGCCGGGTAA) and 806R (GGACTACHVGGGTWTCTAAT) (Caporaso et al., 2011).

### 2.9 Soil metabolomics determination

Example of vacuum freeze-drying: Grinding (30 Hz, 30 s) until it turns into powder with a grinder; gage 0.5 g of the specimen, introducing 1 mL of methanol:isopropanol:water extraction mixture (3:3:2 V/V/V), agitate it at ambient temperature for 3 min, then proceed to sonicate it in a chilled water bath for 20 min; centrifuge at a speed of 12,000 rpm and for 3 min at a temperature of 4°C before moving the supernatant into an injection bottle. Insert 0.020 mL of a specific internal standard (10 μg/mL), use a nitrogen blower to blow dry it, and then freeze it in a freeze dryer; incorporate 0.1 mL of methoxamine pyridine salt (0.015 g/mL), blend at 37°C for 2 h using an oven, followed by mixing in 0.1 mL of BSTFA (with 1% TMCS) and maintaining it at 37°C for half an hour to create a derivatization mixture; and dilute the solution for derivatization to a 1-ml volume, strain it using a 0.22-μm organic phase needle filter, refrigerate it at a temperature of −20°C, and hold for GC–MS detection.

### 2.10 Data analysis

Experimental data analysis was conducted using Microsoft Excel 2010, while the statistical program Statistical Package for the Social Sciences (SPSS, IBM version 24.0) software was employed to perform significant difference analyses. No notable differences or interactions are observed across different years. The interplay between processing and diversity remains evident. Utilize SPSS software for computing the Pearson correlation coefficient. The chart was developed using Sigmallot (version 12.0, Systat Software). We assessed the importance of variations through SPSS software; employed R software for alpha diversity analysis and *t*-test for testing; utilized R software (version 2.15.3) for principal component analysis (PCA) and principal coordinate analysis (PCoA); applied LEfSe software for LEfSe evaluation; executed RDA for environmental factor studies with vegan; the species abundance table and applied graphviz-2.38.0 for network diagram creation; implemented Tax4Fun for predicting microbial species functionality.

## 3 Results

### 3.1 Soybean yields

From 2021 to 2022, notable disparities were observed in the yield of soybeans and the dry matter weight during the continuous cropping of soybeans and the corn–soybean rotation. Persistently engaging in farming markedly lowers the production and weight of soybeans, whereas rotating soybeans for corn considerably boosts their yield and weight (Table 1).

**Table 1:** Effects of continuous cropping and corn–soybean rotation on soybean yield and dry matter weight.

Particular year	Treatment	Yield (kg·hm^−2^)	Dry weight (g plant^−1^)
2021	Crop rotation of soybeans	3127.63 ± 22.63^Aa^	92.43 ± 0.71^Aa^
	Continuous cropping of soybeans	1320.98 ± 6.41^Bb^	60.60 ± 1.56^Bb^
2022	Crop rotation of soybeans	3207.30 ± 46.82^Aa^	95.90 ± 1.39^Aa^
	Continuous cropping of soybeans	1336.81 ± 15.62^Bb^	62.67 ± 0.91^Bb^

Different superscript letters in the same column indicate significant differences at the 0.05 level.

### 3.2 Soybean soil nutrients

During the growth phase of soybean R1, notable differences were observed in soil total nitrogen, total phosphorus, total potassium, total carbon, available phosphorus, nitrate nitrogen, ammonium nitrogen, available potassium, and available phosphorus content across continuous cropping and rotation treatments. Continuous cropping significantly reduced soil total nitrogen, total phosphorus, total potassium, total carbon, available phosphorus, available potassium, nitrate nitrogen, and ammonium nitrogen content. The levels of ammonium nitrogen, available phosphorus, and total phosphorus decreased significantly by 21.15, 17.99, and 34.88%, respectively. Continuous cropping significantly increases the content of available potassium and available phosphorus. During the R6 growth phase of soybean, across continuous cropping and rotation treatments, notable differences were observed in soil total nitrogen, total phosphorus, total potassium, total carbon, available phosphorus, available potassium, nitrate nitrogen, ammonium nitrogen, and available phosphorus. Continuous cropping demonstrated a more significant reduction of total nitrogen, total phosphorus, total potassium, total carbon, available phosphorus, nitrate nitrogen, ammonium nitrogen, and available phosphorus. Notably, ammonium nitrogen and available phosphorus decreasing significantly by 26.45 and 27.30%, respectively. Continuous cropping significantly increases the content of available potassium (Table 2).

**Table 2:** Effects of continuous cropping and corn–soybean rotation on soil nutrients during the growth phases of soybean R1 and R6.

Index	R1		R6	
	Crop rotation	Continuous cropping	Crop rotation	Continuous cropping
TN (g·kg^−1^)	2.18 ± 0.48^Aa^	1.3 ± 0.05^Bb^	2.57 ± 0.55^Aa^	1.74 ± 0.24^Bb^
TP (g·kg^−1^)	3.01 ± 0.48^Aa^	1.96 ± 0.39^Bb^	3.43 ± 0.75^Aa^	2.44 ± 0.22^Bb^
TK (g·kg^−1^)	19.72 ± 0.08^Aa^	18.53 ± 0.15^Bb^	22.65 ± 0.12^Aa^	19.18 ± 0.12^Bb^
TC (g·kg^−1^)	17.87 ± 0.07^Aa^	17.61 ± 0.07^Ab^	17.94 ± 0.05^Aa^	17.25 ± 0.21^Ab^
A-P (mg·kg^−1^)	13.12 ± 0.4^Aa^	10.76 ± 0.45^Bb^	16.01 ± 1.11^Aa^	11.64 ± 0.54^Bb^
AK (mg·kg^−1^)	128.07 ± 1.21^Ab^	132.43 ± 1.27^Aa^	128.55 ± 1.04^Ab^	133.6 ± 2.43^Aa^
NO_3_^—^N (mg·kg^−1^)	1.68 ± 0.15^Aa^	1.13 ± 0.13^Ab^	3.27 ± 0.12^Aa^	2.67 ± 0.2^Ab^
NH_4_^+^-N (mg·kg^−1^)	11.3 ± 0.89^Aa^	8.91 ± 1.33^Bb^	22.54 ± 1.87^Aa^	16.57 ± 1.21^Bb^
DOC (mg·kg^−1^)	30.81 ± 0.12^Aa^	30.37 ± 0.12^Aa^	30.92 ± 0.08^Aa^	29.74 ± 0.36^Aa^
AP (mg·kg^−1^)	10.64 ± 0.47^Bb^	11.12 ± 0.51^Aa^	22.25 ± 1.21^Aa^	20.24 ± 0.71^Bb^
pH	6.07 ± 0.02^Aa^	6.14 ± 0.06^Aa^	5.91 ± 0.02^Aa^	6 ± 0.02^Aa^

TN, total nitrogen, TP, total phosphorus; TK, total potassium.

Different superscript letters in the same column indicate significant differences at the 0.05 level.

### 3.3 Soybean soil enzyme activity

During the growth phase of soybean R1, notable differences were observed in the activities of soil sucrase, catalase, urease, leucine aminopeptidase, and N-acetylglucosamine across continuous cropping and rotation treatments. Continuous cropping led to significant reductions in the activities of soil urease, leucine aminopeptidase, and N-acetylglucosamine, decreasing significantly by 7.7, 16.91, and 29.19%, respectively. Continuous cropping significantly increases the activity of soil sucrase and catalase. During the growth phase of soybean R6, notable differences were observed in the activities of soil sucrase, catalase, urease, leucine aminopeptidase, and N-acetylglucosamine under continuous cropping and rotation treatments. Continuous cropping significantly increased the soil sucrase and catalase content, while significantly reducing the activities of soil urease, leucine aminopeptidase, and N-acetylglucosamine (Table 3).

**Table 3:** Effects of continuous cropping and corn–soybean rotation on soil enzyme activity during the growth phases of soybean R1 and R6.

Growth phase	Treatment	IVE (mg·d^−1^ g^−1^)	S-CAT (mg·d^−1^ g^−1^)	Ure (mg·d^−1^ g^−1^)	LAP (nM·h^−1^ g^−1^)	NAG (nM·h^−1^ g^−1^)
R1	Crop rotation	6.2 ± 0.06^Bb^	11.92 ± 0.66^Bb^	1.42 ± 0.01^Aa^	24.07 ± 1.63^Aa^	17.13 ± 0.86^Aa^
	Continuous cropping	8.43 ± 0.34^Aa^	17.91 ± 0.55^Aa^	1.31 ± 0.1^Ab^	20 ± 0.46^Bb^	12.13 ± 0.77^Bb^
R6	Crop rotation	6.7 ± 0.18^Bb^	12.12 ± 0.6^Bb^	2.11 ± 0.06^Aa^	35.29 ± 1.65^Aa^	28.4 ± 0.83^Aa^
	Continuous cropping	8.72 ± 0.35^Aa^	18.7 ± 0.25^Aa^	1.85 ± 0.04^Ab^	30.49 ± 1.24^Bb^	22.64 ± 0.47^Bb^

IVE, Sucrase; LAP, leucine aminopeptidase; NAG, N-acetylglucosidase; S-CAT, catalase; Ure, urease.

Different superscript letters in the same column indicate significant differences at the 0.05 level.

### 3.4 The impact of corn–soybean rotation on soybean nutrient absorption

#### 3.4.1 Soybean nitrogen accumulation

During the growth phase of soybean R1, notable differences were observed in nitrogen content among soybean stems, leaves, and stems between continuous cropping and corn–soybean rotation treatments. Continuous cropping significantly reduces the nitrogen content of soybean stems, leaves, and petiole, with a decrease rate of 79.22, 60.28, and 59.41%. During the growth phase R6, notable differences were observed in the nitrogen content among soybean stems, leaves, stems, and pods between to corn–soybean rotation treatments. Continuous cropping significantly reduced the nitrogen content of soybean stems, leaves, petioles, and pods, decreasing 57.12, 44.92, 52.75, and 64.94%. During the growth phase R8, notable differences were observed in the nitrogen content of soybean stems, leaves, stems, pods, and grains between continuous cropping and corn–soybean rotation treatments. Continuous cropping significantly reduced the nitrogen content of soybean stems, leaves, petioles, pods, and grains, decreasing significantly by 73.16, 68.21, 61.34, 78.68, and 45.86%, respectively (Table 4).

**Table 4:** Nitrogen content of soybean organs under continuous cropping and corn–soybean rotation treatment (kg·hm^−2^).

Growth phase	Treatment	Stem	Leaf	Petiole	Pod	Grain
R1	Crop rotation	15.69 ± 0.93^Aa^	47.71 ± 1.26^Aa^	9.14 ± 0.25^Aa^	–	–
	Continuous cropping	6.26 ± 0.46^Bb^	18.95 ± 1.15^Bb^	3.71 ± 0.26^Bb^	–	–
R6	Crop rotation	22.90 ± 0.36^Aa^	50.18 ± 3^Aa^	9.46 ± 0.62^Aa^	79.14 ± 5.53^Aa^	–
	Continuous cropping	9.82 ± 1.31^Bb^	27.64 ± 3.65^Bb^	4.47 ± 0.06^Bb^	27.75 ± 1.2^Bb^	–
R8	Crop rotation	11.66 ± 2.2^Aa^	47.56 ± 1.41^Aa^	8.64 ± 0.3^Aa^	47.40 ± 3.85^Aa^	141.43 ± 3.^66Aa^
	Continuous cropping	3.13 ± 0.33^Bb^	15.12 ± 3.82^Bb^	3.34 ± 0.2^Bb^	10.17 ± 1.99^Bb^	76.57 ± 2.07^Bb^

Different superscript letters in the same column indicate significant differences at the 0.05 level.

#### 3.4.2 Accumulation of phosphorus in soybeans

During the growth phase R1, notable differences were observed in the phosphorus content of soybean stems, leaves, and stems between continuous cropping and corn–soybean rotation treatments. Continuous cropping significantly reduces the phosphorus content in soybean stems, leaves, and petioles, with a decrease rate of 54.97, 46.91, and 29.57. During the growth phase R6, notable differences were observed in the phosphorus content of soybean stems, leaves, stems, and pods between continuous cropping and corn–soybean rotation treatments. Continuous cropping significantly reduced the phosphorus content of soybean stems, leaves, petioles, and pods, decreasing significantly by 29.85, 47.13, 42.22, and 54.41%, respectively. During the growth phase R8, notable differences were observed in the phosphorus content of soybean stems, leaves, stems, pods, and grains between continuous cropping and corn–soybean rotation treatments. Continuous cropping significantly reduced the phosphorus content of soybean stems, leaves, petioles, pods, and grains, with a decrease rate of 56.52, 60.70, 24.79, 56.25, and 71.04% (Table 5).

**Table 5:** Phosphorus content in various organs of soybean treated with continuous cropping and corn–soybean rotation (kg·hm^−2^).

Growth phase	Treatment	Stem	Leaf	Petiole	Pod	Grain
R1	Crop rotation	1.71 ± 0.30^Aa^	3.07 ± 0.25^Aa^	1.15 ± 0.36^Aa^	–	–
	Continuous cropping	0.77 ± 0.22^Bb^	1.63 ± 0.20^Bb^	0.81 ± 0.07^Bb^	–	–
R6	Crop rotation	2.01 ± 0.41^Aa^	3.31 ± 0.11^Aa^	1.35 ± 0.08^Aa^	3.29 ± 0.14^Aa^	–
	Continuous cropping	1.41 ± 0.06^Bb^	1.75 ± 0.25^Bb^	0.78 ± 0.04^Bb^	1.50 ± 0.14^Bb^	–
R8	Crop rotation	1.61 ± 0.15^Aa^	3.13 ± 0.08^Aa^	1.17 ± 0.08^Aa^	3.36 ± 0.19^Aa^	8.39 ± 0.10^Aa^
	Continuous cropping	0.7 ± 0.09^Bb^	1.23 ± 0.12^Bb^	0.88 ± 0.13^Bb^	1.47 ± 0.24^Bb^	2.43 ± 0.07^Bb^

Different superscript letters in the same column indicate significant differences at the 0.05 level.

#### 3.4.3 Accumulation of potassium in soybeans

During the growth phase R1, notable differences were observed in potassium content in soybean stems, leaves, and stems between continuous cropping and corn–soybean rotation treatments. Continuous cropping significantly reduces the potassium content in soybean stems, leaves, and petioles, decreasing significantly by 86.37, 53.80, and 72.45%, respectively. During the growth phase R6, notable differences were observed in the potassium content of soybean stems, leaves, stems, and pods between continuous cropping and corn–soybean rotation treatments. Continuous cropping significantly reduced the potassium content of soybean stems, leaves, petioles, and pods, with a decrease rate of 85.35, 44.69, 70.73, and 55.97%. During the growth phase R8, notable differences were observed in the potassium content of soybean stems, leaves, stems, pods, and grains between continuous cropping and corn–soybean rotation treatments. Continuous cropping significantly reduced the potassium content of soybean stems, leaves, petioles, pods, and grains, decreasing significantly by 76.99, 65.44, 45.23, 77.23, and 57.86%, respectively (Table 6).

**Table 6:** Potassium content in various organs of soybean treated with continuous cropping and corn–soybean rotation (kg·hm^−2^).

Growth phase	Treatment	Stem	Leaf	Petiole	Pod	Grain
R1	Crop rotation	10.71 ± 1.19^Aa^	11.71 ± 0.69^Aa^	4.90 ± 0.09^Aa^	–	–
	Continuous cropping	1.46 ± 0.22^Bb^	5.41 ± 0.24^Bb^	1.35 ± 0.25^Bb^	–	–
R6	Crop rotation	13.17 ± 1.64^Aa^	12.33 ± 0.52^Aa^	5.09 ± 0.20^Aa^	29.14 ± 0.43^Aa^	–
	Continuous cropping	1.93 ± 0.33^Bb^	6.82 ± 0.23^Bb^	1.49 ± 0.15^Bb^	12.83 ± 0.29^Bb^	–
R8	Crop rotation	4.91 ± 0.30^Aa^	14.67 ± 0.45^Aa^	4.82 ± 0.015^Aa^	19.54 ± 0.64^Aa^	40.86 ± 0.38^Aa^
	Continuous cropping	1.13 ± 0.^08Bb^	5.07 ± 0.13^Bb^	2.64 ± 0.10^Bb^	4.45 ± 0.08^Bb^	17.22 ± 0.34^Bb^

Different superscript letters in the same column indicate significant differences at the 0.05 level.

### 3.5 Diversity of bacterial and microbial communities in soybean soil

For the 16S rRNA sequencing data, 1,539,815 reads were acquired from 18 distinct samples. After splicing and sifting through the double-ended reads, a cumulative count of 1,523,222 combined reads was formed. Every specimen contains a minimum of 77,515 reads plus 76,575 combined reads. The sequences of all samples revealed the presence of 17,136 operational taxonomic units (OTUs). Continuous cropping treatments, namely, LCB, LCR (continuous cropping), LCP (endosphere bacterial communities in continuous soybean cropping), and crop rotation (LRCB [soil bacteria in the rhizosphere of corn–soybean rotation], LRR [soil bacteria on the rhizoplane of corn–soybean rotation], LRP [soil bacteria in endosphere of corn–soybean rotation], chao1, ace, and OTUs), experienced a progressive decline (Table 7).

**Table 7:** Rotation and continuous cropping treatments on soybean soil bacteria diversity.

Treatment	Observed_Species	Shannon	Simpson	Chao1	ACE	Goods_Coverage	PD_Whole_Tree
LCR	2881.33 ± 222.05^Aa^	8.97 ± 0.29^Aa^	0.99 ± 0.00A^a^	3216.11 ± 279.60^Aa^	3260.1 ± 274.37^Aa^	0.99 ± 0.00^Aa^	213.22 ± 13.47^Aa^
LCB	2973.33 ± 205.79^A^a	9.24 ± 0.10^Aa^	0.99 ± 0.00A^a^	3284.49 ± 210.47^Aa^	3345.56 ± 231.47^Aa^	0.99 ± 0.00^Aa^	249.07 ± 53.92^Aa^
LCP	2808.67 ± 32.96A^a^	9.04 ± 0.09^Aa^	0.99 ± 0.00A^a^	3119.45 ± 51.46^Aa^	3162.71 ± 41.15^Aa^	0.99 ± 0.00^Aa^	226.37 ± 23.15^Aa^
LRR	2905.33 ± 99.68A^a^	9.18 ± 0.08^Aa^	0.99 ± 0.00A^a^	3224.4 ± 163.33^Aa^	3244.16 ± 114.48^Aa^	0.99 ± 0.00^Aa^	248.11 ± 32.33^Aa^
LRCB	2884.33 ± 71.16A^a^	9.09 ± 0.11^Aa^	0.99 ± 0.00A^a^	3207.61 ± 70.94^Aa^	3260.3 ± 68.08^Aa^	0.99 ± 0.00^Aa^	237.13 ± 39.57^Aa^
LRP	2,706 ± 595.08A^a^	7.77 ± 2.53^Aa^	0.93 ± 0.00A^a^	3054.13 ± 660.42^Aa^	3134.25 ± 661.38^Aa^	0.99 ± 0.00^Aa^	227.63 ± 56.82^Aa^

OTUs: It is a group of individuals with similar DNA sequences; LCR: soil bacteria on the rhizoplane of soybean continuous cropping; LCB: soil bacteria in the rhizosphere of soybean continuous cropping; LCP: endosphere bacterial communities in continuous soybean cropping; LRR: soil bacteria on the rhizoplane of corn–soybean rotation; LRCB: soil bacteria in the rhizosphere of corn–soybean rotation; LRP: soil bacteria in endosphere of corn–soybean rotation.

Different superscript letters in the same column indicate significant differences at the 0.05 level.

### 3.6 Microbial community structure of soybean root bacteria

**Figure 1:**
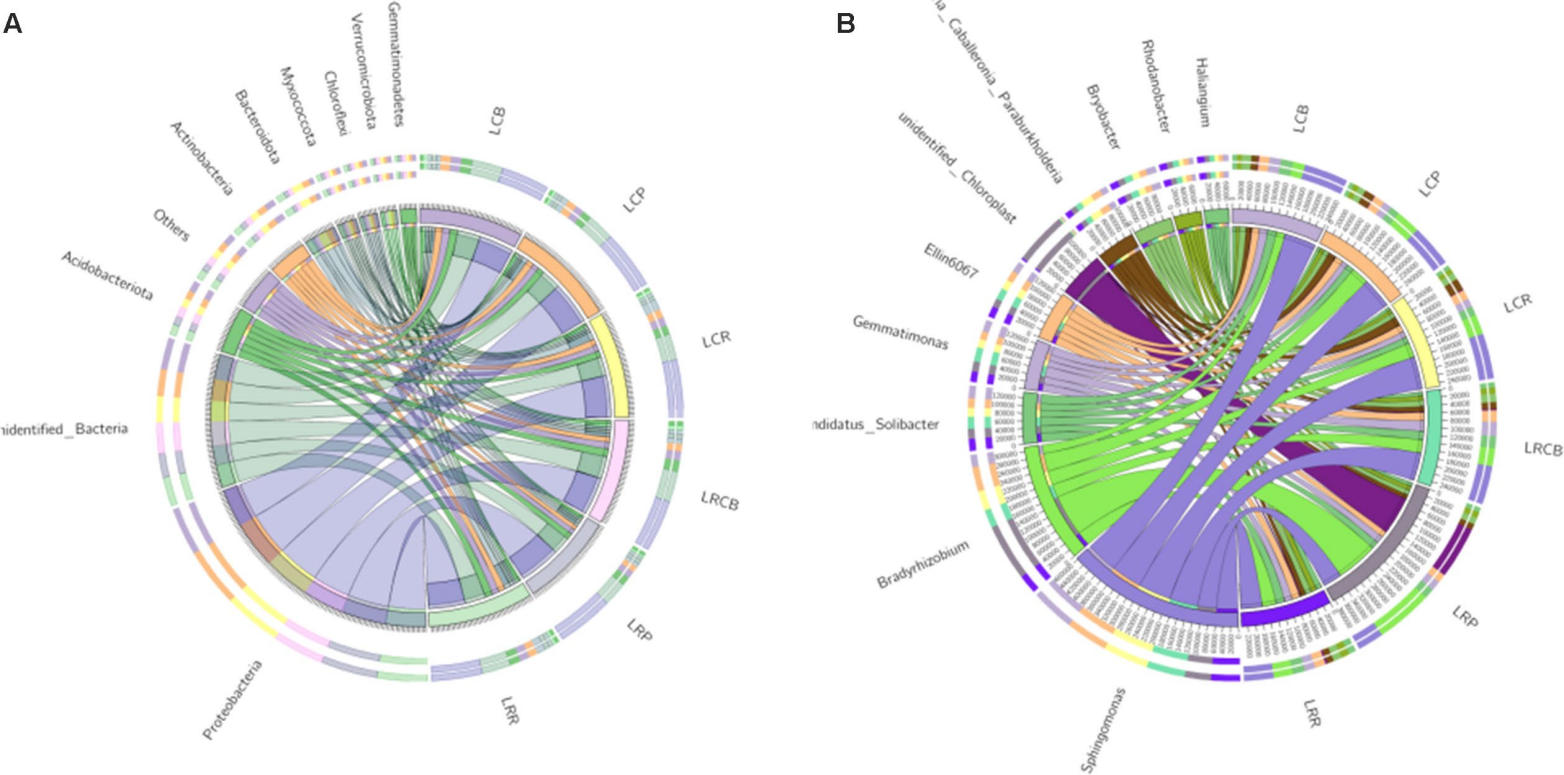
Composition of bacterial and microbial communities in different ecological niches under **(A)** soybean continuous cropping and **(B)** corn–soybean rotation treatments.

Within ecological phylum, other classifications included OTUs predominantly linked to Proteobacteria, unidentified_Bacteria, Acidobacteriota, Actinobacteria, Bacteroidota, Myxococcata, Chloroflexi, Verrucomicrobiota, Gemmatimonades, and Cyanobacteria, constituting 85.5, 88.7, 88.4, 87.4, 87.4, and 89.9% of the OTUs in each category (Figure 1A). Among all sampled groups, Proteobacteria stands out as the most common group. Within the genus classification, along with other categories, OTUs in each sample group were predominantly associated with Sphingomonas, Bradyrhizobium, Burkholderia Caballeronia Paraburkholderia, Ellin6067, Variovorax, Candidatus_Solibacter, Gemmatimonas, Sphingobacteria, Bryobacter, and Novosphingobium. Notably, at the genus level, the prevalence of Sphingobacteria within the three ecological niches resulting from continuous cropping treatment markedly surpassed those in the equivalent ecological niche associated with crop rotation treatment (Figure 1B).

### 3.7 Enrichment/consumption of OTUs in soybean root bacteria microbiome

To better understand bacterial OTUs influenced by various ecological environments, we performed an analysis of OTUs’ varied abundance using negative binomial distribution techniques. With rhizosphere soil serving as a control group, a *p*-value below 0.01 was established. The number of bacterial OTUs enriched in rhizoplane and endosphere of continuous cropping exceeded that in rotation treatments (LCR: 141, LCP: 98; LRR: 97; LRP:71). Bacterial OTUs ingested in rhizoplane and endosphere of continuous cropping were less compared to crop rotation treatments (LCR: 47; LCP: 56; LRR: 68; LRP: 65). Apart from a lesser amount of bacterial OTUs on rhizoplane than in endosphere of continuous cropping, bacterial OTUs enriched and ingested on rhizoplane of both methods surpassed those in endosphere. Under varied treatments, the increase and utilization of bacterial OTUs occur in multiple root ecological environments. During continuous cropping, a convergence occurred between 34.75 and 50% of OTUs found on both rhizoplane and endosphere. In the OTUs eaten, there was an intersection of 25.53 and 21.43%. In terms of crop rotations, the overlap was 17.53 and 23.94% between OTUs on rhizoplane and endosphere. For consumption rates, these OTUs demonstrated 39.71 and 41.54% overlap. This suggests that soil bacteria have the ability to gather from the rhizosphere, rhizoplane, and endosphere to inhabit plant roots.

**Figure 2:**
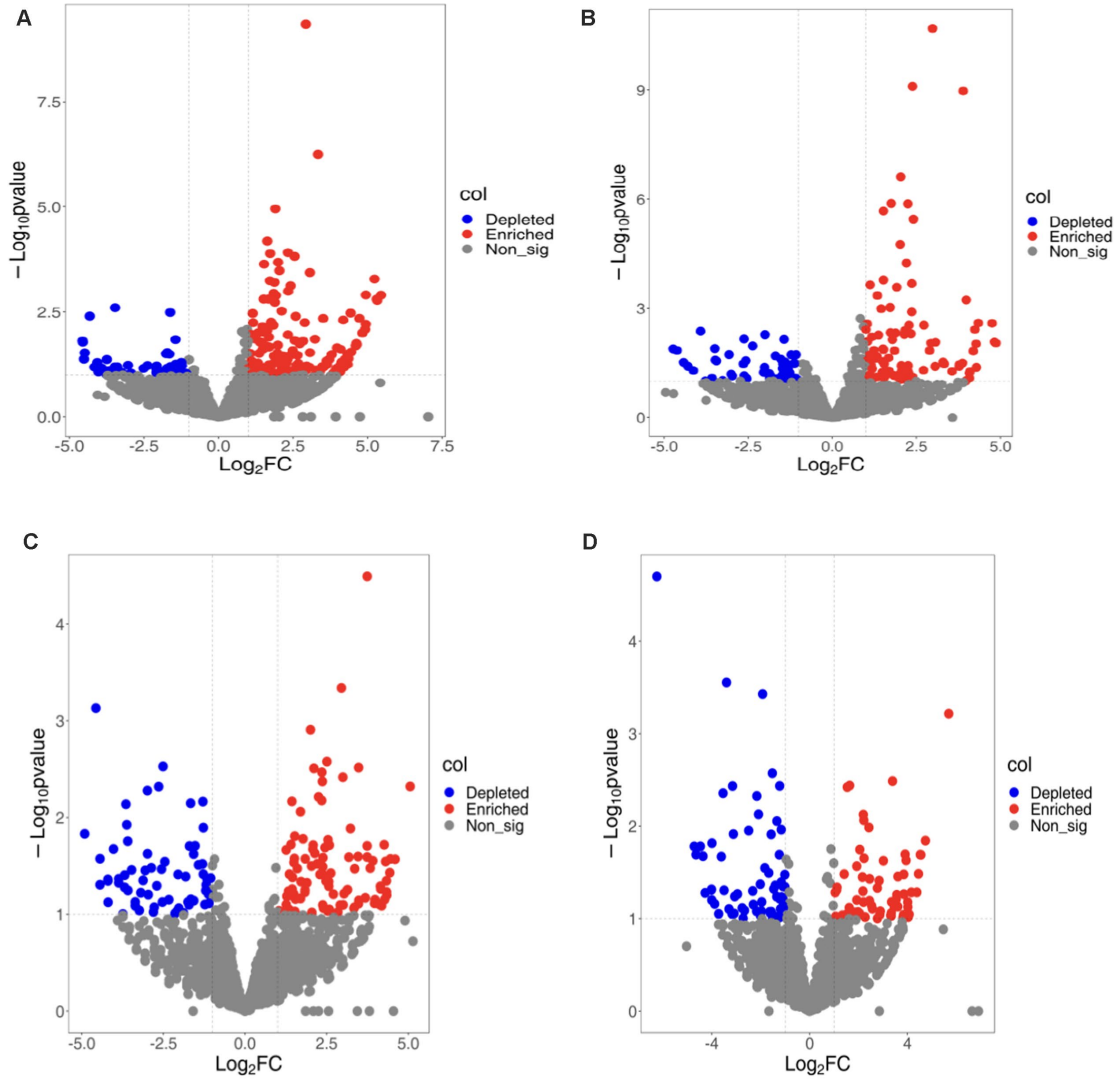
Bacterial OTUs enriched/consumed in different ecological niches under soybean continuous cropping and corn–soybean rotation treatments. **(A)** Continuous cropping rhizoplane; **(B)** continuous cropping endosphere; **(C)** rotation rhizoplane; and **(D)** rotation endosphere.

There is a diversity in the count and varieties of bacterial OTUs across various treatments and ecological niches. The diversity and variety of bacterial OTUs, enriched and ingested across diverse treatments and ecological habitats, also differ. OTUs predominantly found on rhizoplane of continuous cropping and rotation are mainly Variovorax Allorhizobium, Burkholderia, Stenotrophomonas, Beijerinckiaceae, Mucilaginibacter. Conversely, root OTUs on this rhizoplane include Bradyrhizobium Bosea, Mucilaginibacter, Sphingobacterium, Enterobacter, Cupriavidus. Endosphere consumed OTUs are primarily Rhodobacteraceae, Flavisolibacter, Pedosphaeraceae. Endosphere consumed OTUs that predominantly consist of Haliangiaceae Nitrososphaeraceae, Chitinophagacteria. Endosphere exposed OTUs are Sphingopyxis Chitinophaga, Pedobacter, Chryseobacteria Subgroup_10, and Sandaracinaceae, while those consumed are mainly Candidatus-Nitrocomicus LWQ8 and Pseudoxanthas. Within the rhizoplane of continuous cropping are mainly Edaphobulum, Bodovdellibrio, and Hymenacteobacteria (Figure 2).

### 3.8 Co-occurrence network of soybean soil bacteria and microorganisms

**Figure 3:**
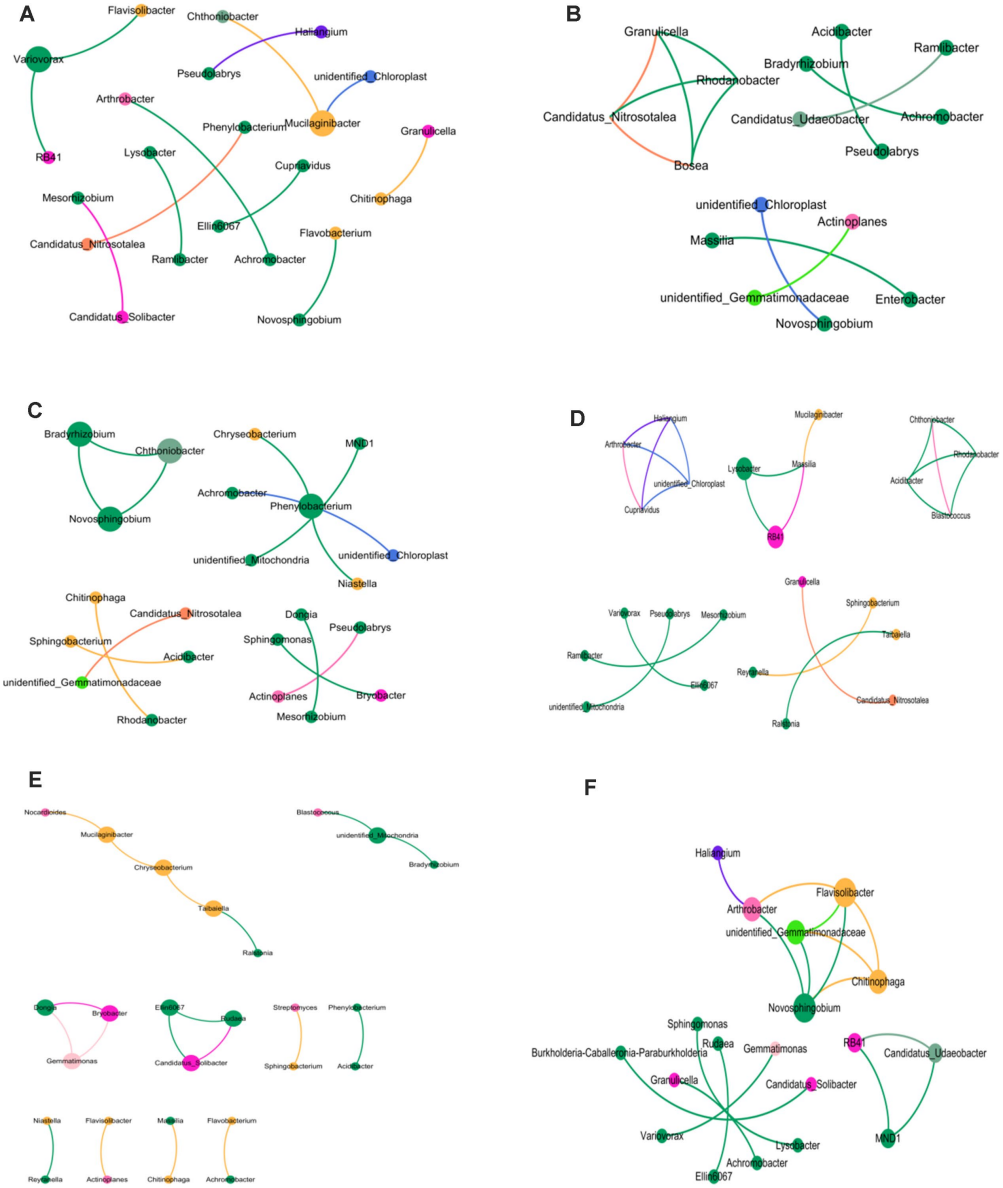
Cooccurrence network of bacterial genera in different ecological niches under soybean continuous cropping and corn–soybean rotation treatments. **(A)** Soil bacteria in the rhizosphere of soybean continuous cropping; **(B)** soil bacteria on the rhizoplane of soybean continuous cropping; **(C)** endosphere bacterial communities in continuous soybean cropping; **(D)** soil bacteria in the rhizosphere of corn–soybean rotation; **(E)** soil bacteria on the rhizoplane of corn–soybean rotation; and **(F)** soil bacteria in the endosphere of corn–soybean rotation.

This research involved creating six co-occurrence networks to demonstrate the variations in soil bacterial populations across rhizosphere, rhizoplane, and endosphere, considering crop rotation and continuous cropping techniques. Within the bacterial framework, the trio of ecological niche system networks in crop rotation soil surpasses those in continuous cropping treatment, with both Edges and Shortest paths being more expansive than continuous cropping treatment. An increased clustering coefficient correlates with greater node clustering intensities within the co-occurrence network. There’s a diminishing clustering coefficient from the rhizosphere, through rhizoplane to endosphere in crop rotation, while an increase occurs when continuous cropping is applied. Within the aforementioned six co-occurrence networks, Proteobacteria, Acidobacteriota, and Bateroidota emerged as predominant phyla, whereas Cyanobacteria were exclusively present in continuous cropping scenarios (Figure 3).

### 3.9 Dominant bacteria in soybean root system microbiome

**Figure 4:**
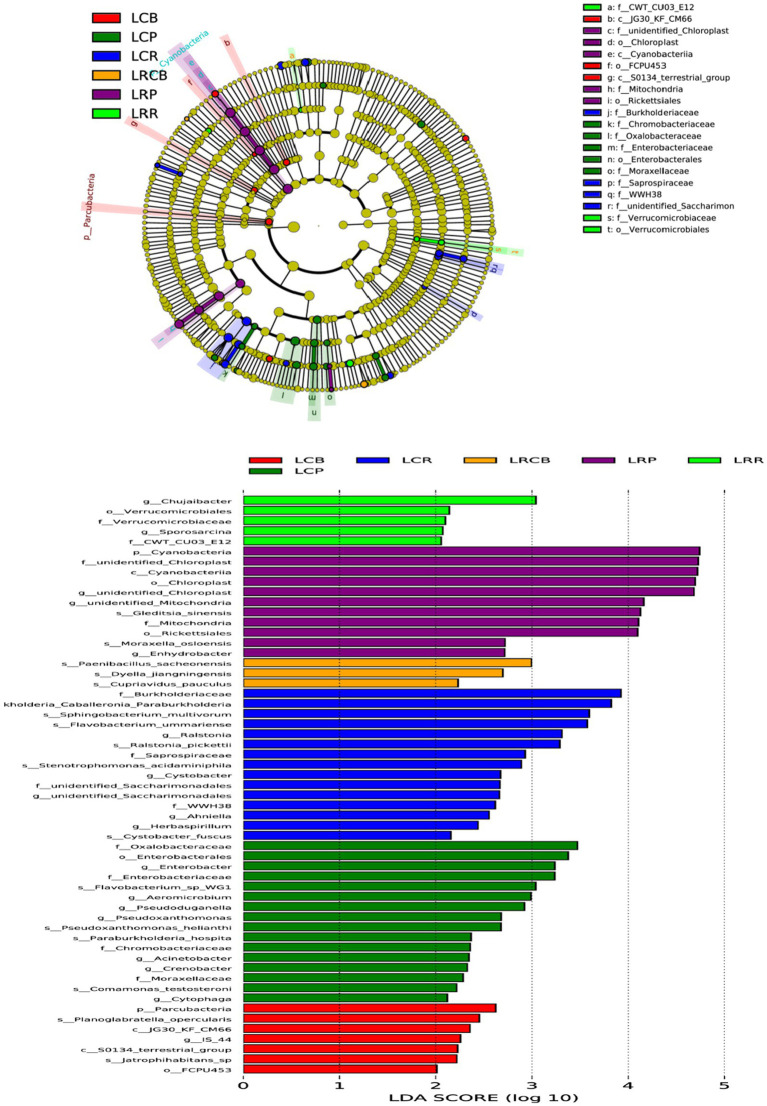
Based on LEfSe analysis of dominant bacterial communities in different ecological niches of soybean continuous cropping and corn–soybean rotation root systems. LefSe analysis is LDA effect size analysis, which is an analysis tool for high-dimensional data (genes, pathways, microbial taxonomic units, etc.). It can compare two or more groups and find biomarkers with statistical differences between groups.

For discerning bacterial populations that vary significantly in presence between continuous crops and rotating crops, linear discriminant analysis (LDA) effect size (LEfSe) was employed as a biomarker analysis technique (Figure 4). The implementation of crop rotation enhanced the proliferation of Leuciscinae genus, Verrucomicrobiota order, and Bacteroides genus bacteria in root soil; boosted the presence of spore-producing bacteria, Dictynia species, and copper-affirming bacteria on rhizoplane; and enhanced reproductive capabilities in the soil’s Cyanobacteria, chloroplast and mitochondrial-associated bacteria, rickettsia, Oslo Moraxeales, and root-based water-dwelling bacteria. Conversely, consistent cropping enhanced root soil growth with Burkholderia, Sphingomonas, Bacteroidetes, Ralstonia, Ustilaginaceae, Xanthomonas, Ascomycota, Anila, and Spirogyra; elevated levels of Candida phylum, Planoglabratellatoarchecularis, and Jatrophihabitans bacteria in the rhizosphere and increased the spread of Oxalibacter, Enterobacteriaceae, Flavobacterium, Aeromonas, Pseudorhizobium, Burkholderia, Klebsiella, Moraceae, and Phagocytosis endosphere.

### 3.10 Microbial community function of soybean soil bacteria

**Figure 5:**
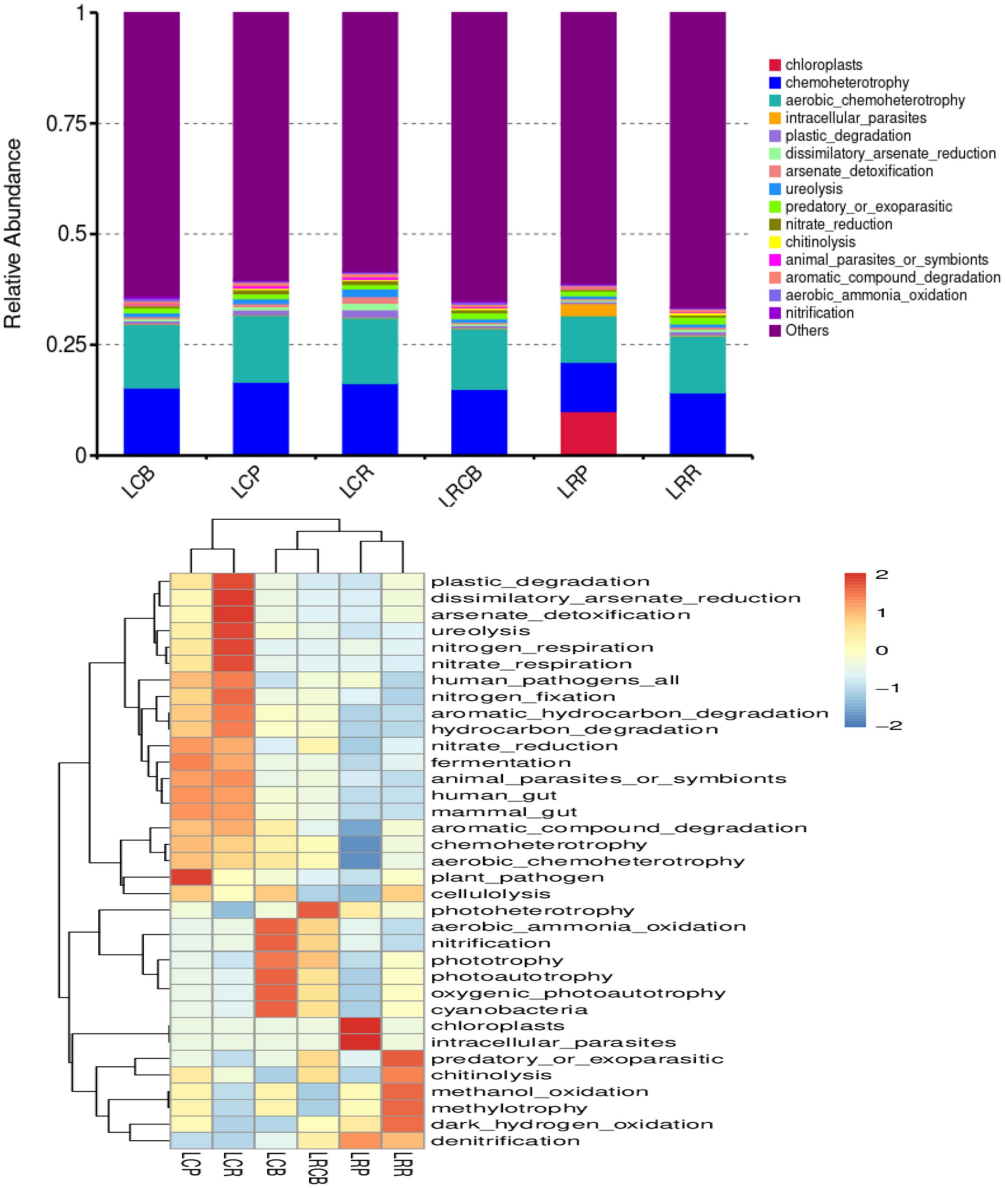
Classification and annotation heatmap of soil bacterial functions in soybean continuous cropping and corn–soybean rotation.

Alterations in bacterial populations additionally impact the functioning of bacteria. Consequently, the outcomes of the experiments were scrutinized using the 16S FAPROTAX functional forecast. A bar graph displaying the top 15 predicted functional classifications for each genus, alongside a heatmap showcasing the top 35 bacterial functional classifications for the same genus, reveals notable alterations in bacterial activities across various ecological sections of the endosphere, influenced by crop rotation and continuous cropping practices. Alterations in bacterial populations within crop rotation roots result from the bacteria’s altered control over chloroplasts, intracellular parasites, and additional activities. The role of soil bacteria in managing predator or parasite infestations, the breakdown of chitin, methanol oxidation, methyl nutrition, and dark hydrogen oxidation in the rhizoplane of crop rotations. There have been notable shifts in the photosynthesis (energy) heterotrophic and photosynthetic activities of the soil bacteria at the crop’s rhizoplane. Similarly, the harmful effects of bacteria and plants in the endosphere of continuous cropping have turned irregular. Abnormal alterations have been observed in the processes of nitrate and nitrogen respiration, urea breakdown, arsenate detoxification, and the diminution of alienated arsenate in bacteria at the rhizosphere of continuous cropping plants; Moreover, these processes show unusual shifts in the aerobic oxidation of ammonia and nitrification capabilities of rhizoplane bacteria in such cropping scenarios (Figure 5).

### 3.11 Potential pathogenic and beneficial bacteria in soybean bacterial microbiome

**Figure 6:**
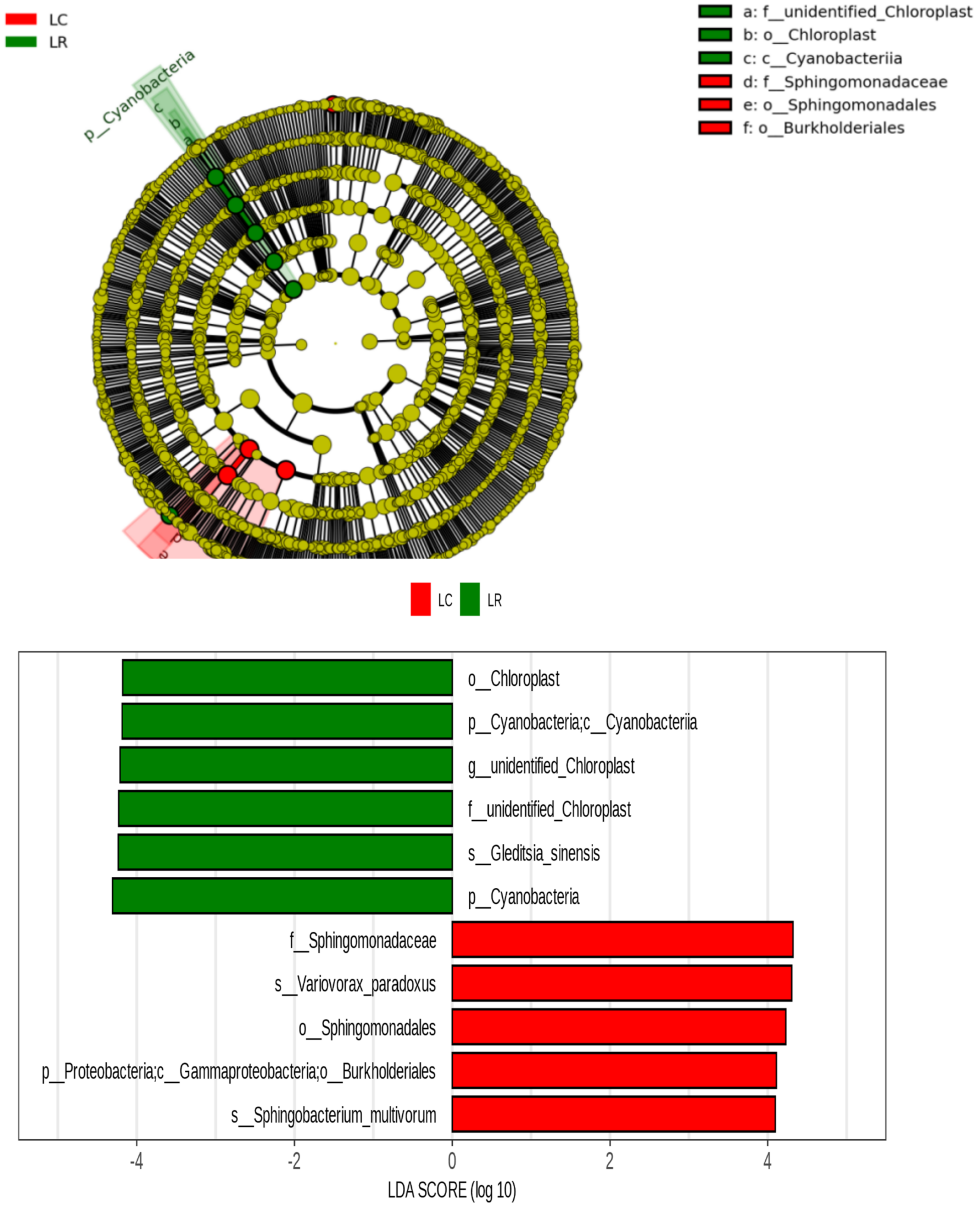
LEfSe analysis of different bacteria in soybean continuous cropping and corn–soybean rotation treatment.

By examining the considerable variations in the relative abundance of various root soil ecosystems due to soybean rotation and continuous cropping, along with predictions of bacterial function and a review of existing literature, we screened 224 prospective pathogenic bacteria and 20 beneficial bacteria. There was a notable rise in the prevalence of 17 harmful bacteria, such as Enterobacter, Cupriavidus, Burkholderia, and Cupriavidus, in endosphere areas of sustained crop growth; 16 such pathogenic bacteria, including Variovorax and Comamonadaceae, saw a substantial increase in endosphere of continuous cropping; advantageous bacteria found in crop rotation treatments are notably more abundant. The LAFSe study was conducted on bacterial OTUs common to the trio of root chambers in both treatments. The continuous cropping treatment notably increased Burkholderiales’ presence, whereas Cyanobacteria demonstrated a notable increase in the crop rotation treatment (Figure 6).

### 3.12 Soil environmental factors bacterial interaction relationship

**Figure 7:**
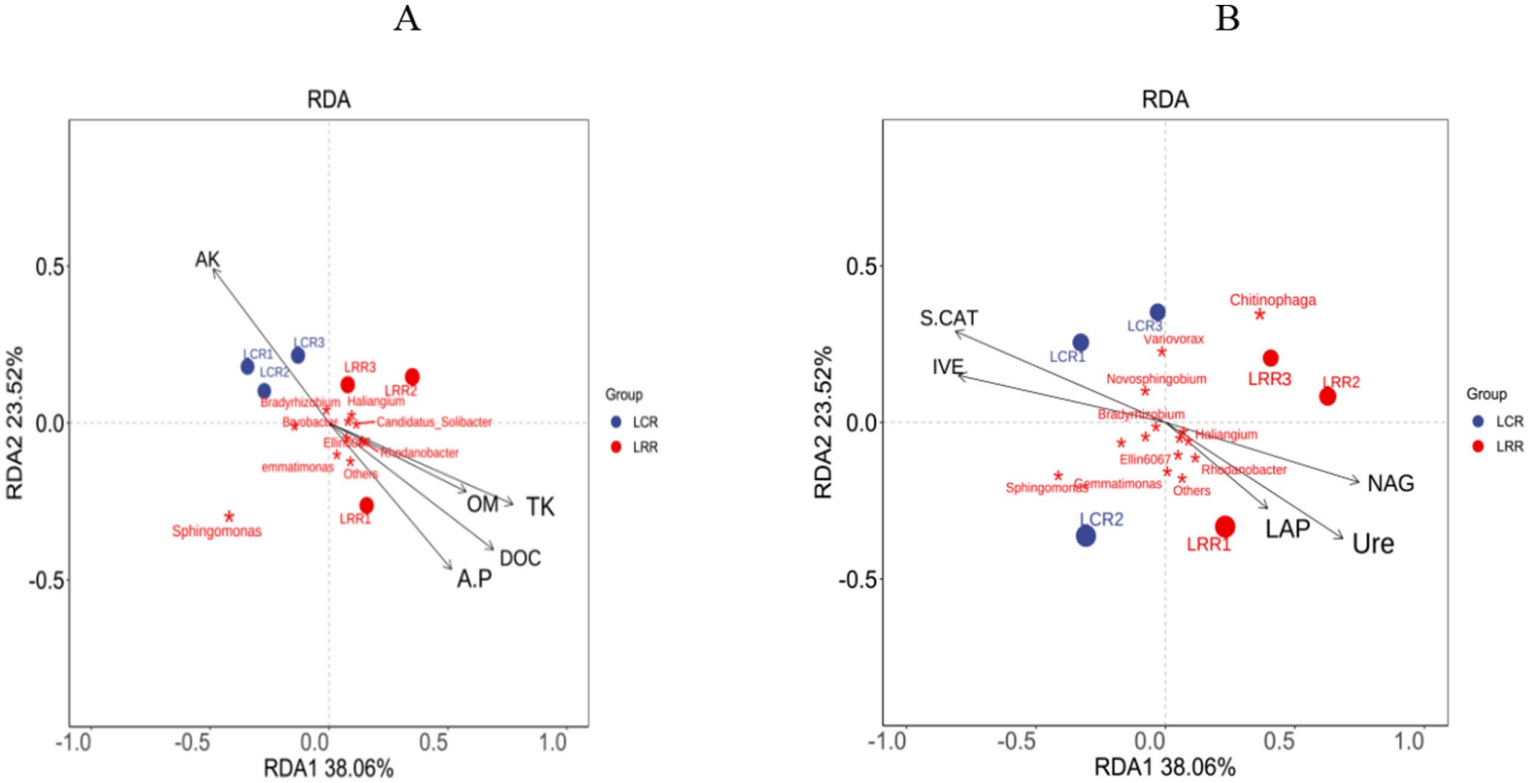
RDA analysis of rhizosphere environmental factors and soil microbial genus levels in crop rotation and continuous cropping treatments. **(A)** Soil physical and chemical factors and soil bacterial RDA analysis; **(B)** soil enzyme and soil bacterial RDA analysis. AK, quick-acting potassium; A.P., Quick-acting phosphorus; OM, organic matter; DOC, organic carbon; TK, total potassium; IVE, sucrase; S-CAT, catalase; Ure, urease; LAP, leucine aminopeptidase; NAG, N-acetylglucosidase; LRR crop rotation; LCR, continuous cropping.

Within the bacterial RDA distribution framework, the first two RDA axes represent 38.1% (RDA1) and 23.5% (RDA2), respectively. These factors, namely, accessible potassium, accessible phosphorus, organic material, carbon, overall potassium, sucrase, catalase, urease, leucine aminopeptidase, and N-acetylglucosamine, are key soil enzymes in influencing the comparative prevalence of Rhodobacter, Bradyrhizobium, Sphingomonas, Variovorax, Burkholderia, Sphingomonas, Gemmatimonas, and the genera Chitinophaga. In these OTUs’ studies, LRR therapy demonstrated significant links among urease, leucine aminopeptidase, N-acetylglucosamine, available phosphorus, organic matter, organic carbon, overall potassium, and Rhodobacter and Gemmatimonas species. Conversely, with LCR therapy, there was a marked positive correlation among available potassium, sucrase, catalase, and Bradyrhizobium, a gradually proliferating rhizobia genus (Figure 7).

### 3.13 Soybean soil metabolites

Examine the varying metabolites in continuous farming and crop rotation practices to identify the fundamental differential metabolites. Notable disparities exist in the levels of 18 metabolites when comparing crop rotation with continuous cropping soil conditions. In comparison to the crop rotation soil, there was a notable decrease in sucrose, sitosterol, benzyl alcohol, malonic acid, and hydroxyurea levels in the soil of continuous cropping. In contrast, there was an increase in the levels of stigmasterol, brassinosterol, eicosanoic acid, octadecanamide, pentadecanoic acid, Galactose, sorbitol, inositol, 2,3-dihydroxypropyl dihydrogen phosphate, arabitol, 1,5-pentanediamine, glycerol, carbonic acid, and glycerol (Table 8).

**Table 8:** Classification of core differential metabolites in soybean continuous cropping and corn–soybean rotation soil.

Index	Formula	Compounds	Class I	CAS	Type
S_TMSMW0490	C29H48O	Stigmasterol 2	Alcohol	83-48-7	Up
S_TMSMW0083	C20H40O2	Eicosanoic acid	Acid	506-30-9	Up
S_TMSMW0762	C18H35NO	(Z)-9-Octadecenamide 1	Amine	301-02-0	Up
S_TMSMW0595	C15H30O2	Pentadecanoic acid 2	Acid	1002-84-2	Up
S_TMSMW0216	C7H14O7	Galactoheptulose 2	Carbohydrate	3019-74-7	Up
S_TMSMW0588	C6H14O6	Sorbitol 1	Carbohydrate	50-70-4	Up
S_TMSMW0215	C6H12O6	Myo-inositol 3	Alcohol	87-89-8	Up
S_TMSMW0372	C3H9O6P	2,3-Dihydroxypropyl dihydrogen phosphate	Acid	57-03-4	Up
S_TMSMW0284	C5H12O5	D-Arabinitol 2	Carbohydrate	488-82-4	Up
S_TMSMW0710	C5H14N2	1,5-Pentanediamine	Amine	462-94-2	Up
S_TMSMW0161	C3H8O3	Glycerin	Alcohol	56-81-5	Up
S_TMSMW0035	CH2O3	Carbonic acid 2	Acid	463-79-6	Up
S_TMSMW0230	C12H22O11	Sucrose	Carbohydrate	57-50-1	Down
S_TMSMW0287	C7H14O6	D-Pinitol	Alcohol	10284-63-6	Down
S_TMSMW0265	C7H8O	Benzyl alcohol	Alcohol	100-51-6	Down
S_TMSMW0151	C3H4O4	Propanedioic acid 2	Acid	141-82-2	Down
S_TMSMW0144	CH4N2O2	Hydroxyurea	Nitrogen compounds	127-07-1	Down

**Figure 8:**
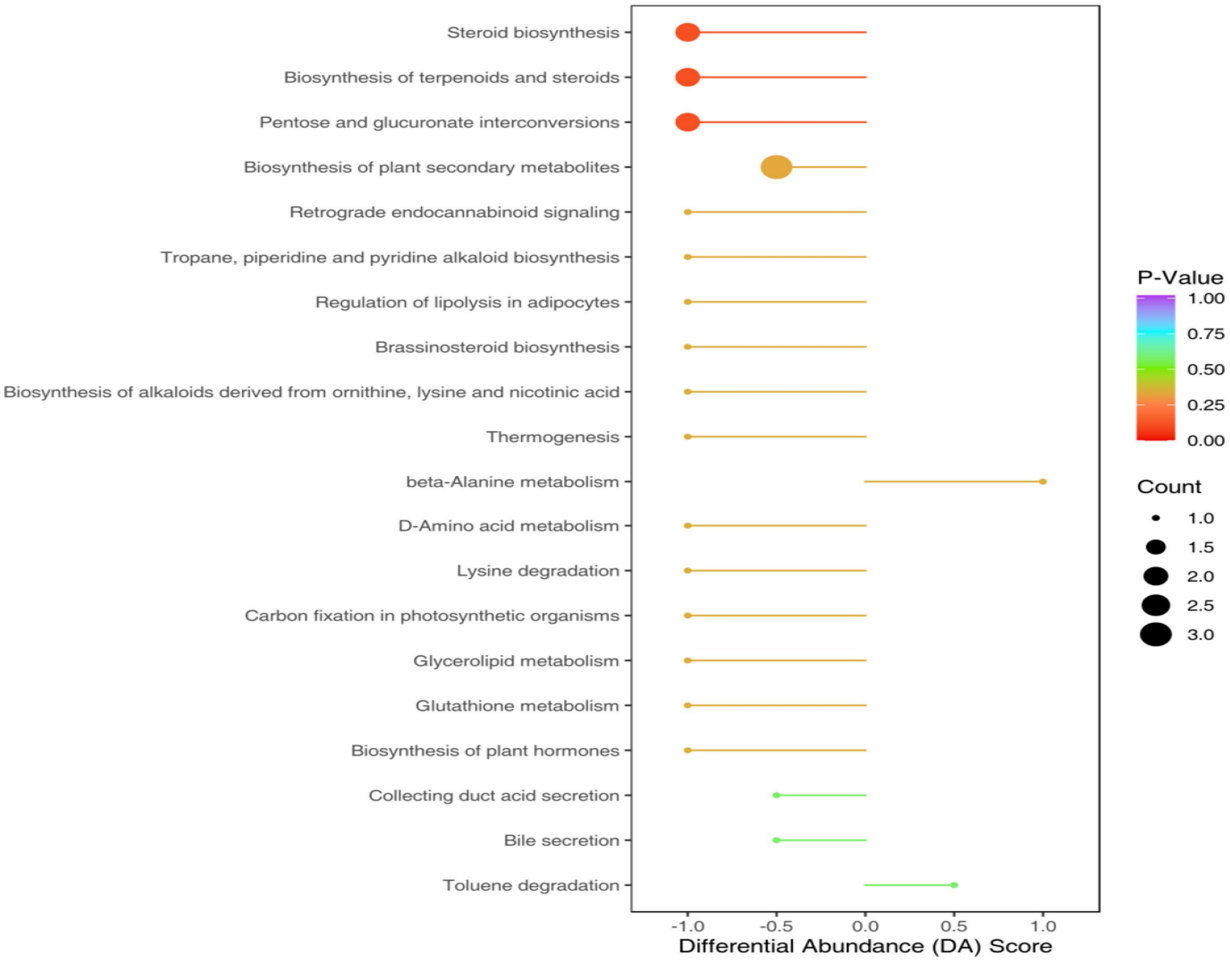
Pathway diagram of differential metabolite annotation between soybean continuous cropping and corn–soybean rotation.

The KEGG annotation process identified that the aforementioned differential metabolites were categorized into 38 functional pathways under four primary functional classifications. Variations in metabolic processes resulted in altered metabolic routes. Within this group, metabolic pathway pathways were noted for having the most diverse metabolite annotations in the primary functional classification, succeeded by unsaturated fatty acid biosynthesis and secondary metabolite biosynthesis pathways, also featuring distinct metabolite annotations (Figure 8).

### 3.14 The connection between the differential metabolites in soiled soybean cores and the bacteria in soil cores

**Figure 9:**
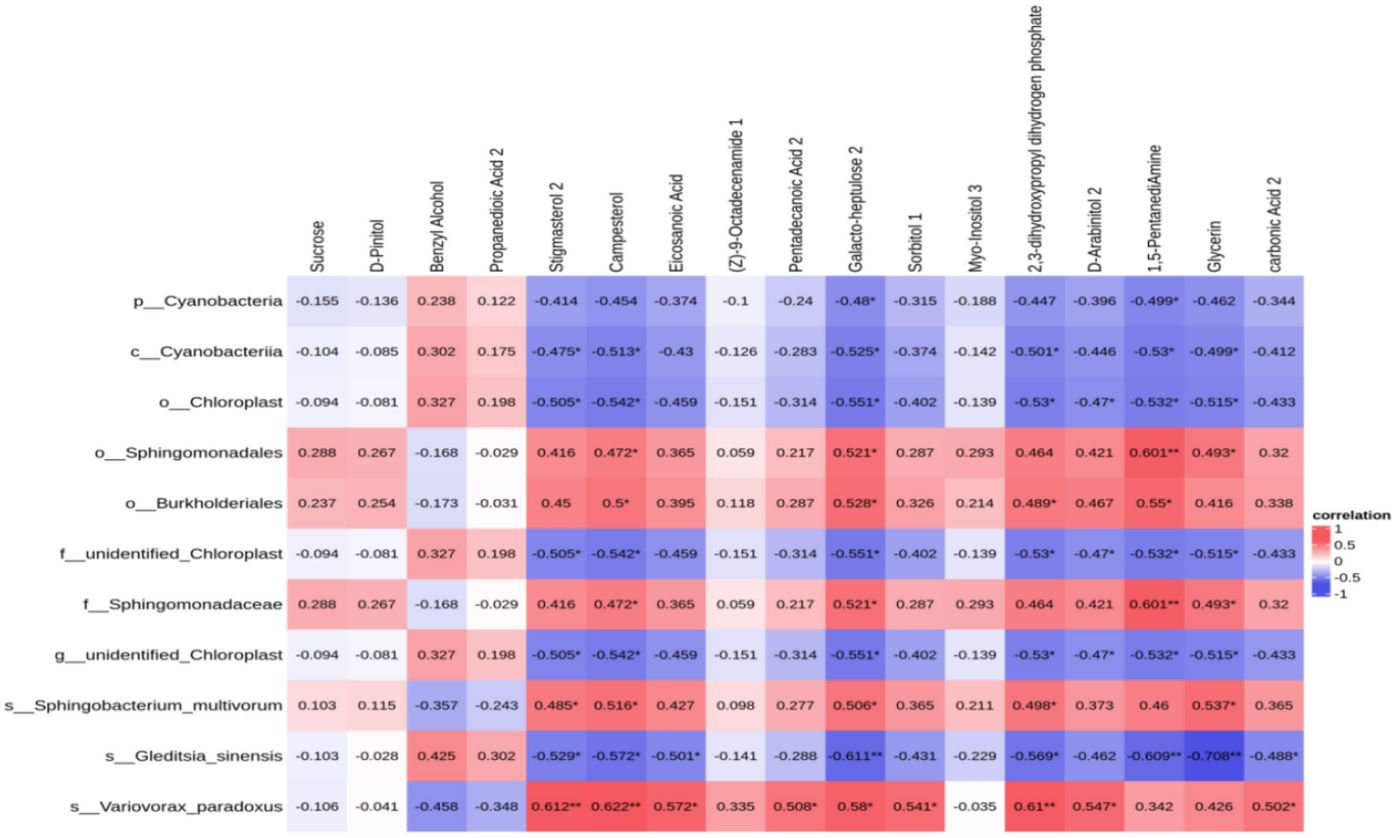
Heat map of the correlation between soil core differential metabolites and soil core differential bacteria in soybean continuous cropping and corn–soybean rotation treatments.

Upon identifying distinct bacteria and performing a correlational analysis between varied bacteria and metabolites, 11 varied bacteria demonstrated a significant association with specific metabolites: Sphingomonadaceae had a notable connection with 1,5-Pentanadiamine (*c* = 0.601, *p* = 0.008), Galactoheptulose (*c* = 0.521, *p* = 0.027), Glycine (*c* = 0.493, *p* = 0.038), and Campesterol (*c* = 0.472, *p* = 0.048); Cyanobacteria demonstrated a significant correlation with 1,5-Pentanadiamine (*c* = −0.530, *p* = 0.024), Galactoheptaose (*c* = −0.525, *p* = 0.025), 2,3-dihydroxypropyl dihydrogen phosphate (*c* = −0.501, *p* = 0.034), Glycine (*c* = −0.499, *p* = 0.035), and Stigmasterol (*c* = −0.475, *p* = 0.046); There is a notable association of chloroplast with several chemicals: Galactoheptaose (*c* = −0.551, *p* = 0.018), 1,5-Pentanadiamine (*c* = −0.532, *p* = 0.023), 2,3-dihydroxypropyl dihydrogen phosphate (*c* = −0.530, *p* = 0.024), Glycine (*c* = −0.515, *p* = 0.029), and Galactoheptaose (*c* = −0.551, *p* = 0.018), while Burkholderiales demonstrated a significant connection with 1,5-Pentanadiamine (*c* = 0.550, *p* = 0.018). The aforementioned findings reveal a distinct link between soil bacteria and their metabolites, with a clear interaction that facilitates and coordinates soybean growth (Figure 9).

## 4 Discussion

The hurdles in uninterrupted soybean farming stem from the combined effect of the soybean and various elements within its soil microenvironment. Unusual variations in soil microbe communities and their variety significantly contribute to continuous challenges in soybean crop (Liu et al., 2022). The soil constitutes a substantial organism of life, with microorganisms playing a crucial role in sustaining soil-based life. Effective collaboration between crops and soil microbes is crucial for achieving sustainable development in agriculture (Franco-Correa and Chavarro-Anzola, 2016). Research indicates that continuous cropping of soybean plants may lead to alterations in the functioning and variety of soil bacteria, consequently boosting the levels and prevalence of harmful bacteria in the soil of soybean roots, which results in persistent challenges in soybean farming (Rao et al., 2021). According to Bulgarelli and colleagues, plant soil microbes comprise dual components. The initial segment consists of microorganisms thriving beyond plant roots, while the latter segment comprises those inhabiting within the endosphere. Nonetheless, the significance of microbial diversity, composition, and the interaction between the rhizoplane and the rhizosphere in bridging the endosphere and external segments remains ambiguous (Edwards et al., 2015). The research employed both soil 16S and non-specific metabolomics sequencing techniques to explore the distribution of soil bacteria within the rhizosphere, rhizoplane, and the endosphere during soybean crop and rotational processes, including soil metabolite effects, aiming to enhance understanding of the barriers in soybean continuous cropping.

### 4.1 Assembly rules of soybean soil bacteria

The root microbiome may either directly or indirectly enhance the healthy proliferation of hosts (Glick, 2012; Miller et al., 2018), and additional research has established that a combination of plants and plant root microbial communities creates an ecosystem where microbes thrive through diffusion, diversification, selection, and drift. The intricacy of their interactions dictates the level of engagement between host organisms and microbes. Scholars segmented the root microbiota into rhizosphere, rhizoplane, and endosphere, varying the root system’s spatial extents, observing diverse microbial communities’ compositions and diversity across these chambers (Edwards et al., 2015). This study found that during continuous cropping and rotating crop treatments, bacterial population alterations varied among the three root compartments within the rhizosphere, rhizoplane, and endosphere. In the gate-level analysis, there was a rise in Proteobacteria numbers from the rhizosphere to the endosphere and then to the endosphere across both treatments. Conversely, Chloroflexi and Actinobacteriota decreased from the rhizoplane to the rhizosphere. Within community levels, the prevalence of Chitinophaga, Burkholderia, Caballeronia, Paraburkholderia, Candidatus_Solibacter, Rhodanobacter, Ramlibacter, Bacillus, Variovorax, Sphingobacterium, and Gemmatimonas is decreasing, transitioning from the rhizosphere, rhizoplane, to endosphere. The findings suggest that soil bacteria inhabit various root chambers via a process of either active or passive diffusion, natural selection (Miller et al., 2018). During continuous cropping rotation, the count of bacterial OTUs on rhizoplane and endosphere of continuous cropping exceeds the number consumed; however, bacterial OTUs ingested on rhizoplane and endosphere continuous cropping are less than crop rotation treatment. In contrast to crop rotations, the regimen of continuous cropping resulted in an increased count of bacterial OTUs concentrated in the trio of root chambers. Following the review of pertinent studies and amalgamation of bacterial function forecasts, the analysis revealed that under consistent cropping therapy, majority of OTUs in the trio of root chambers comprised pathogenic bacteria such as Variovorax, Nitrosotalea, Enterobacter, among others. The findings suggest that persistent cropping enhances the prevalence of pathogenic bacteria in the soil, exhibiting a distinct pattern of selective colonization from the rhizoplane to the rhizosphere. Bacteria-causing soil diseases significantly risk crop yields, the natural environment, and human wellbeing. A surge in the prevalence of pathogenic bacteria in soil could trigger diseases in crops, resulting in diminished agricultural outputs (Ye et al., 2019). Different from continuous cropping, advantageous soil bacteria, Bacillus, demonstrated enrichment across all three root chambers during the crop rotation process. The significant role of Bacillus in soil conditions enhances the nutrient use of crop roots and bolsters resistance to stress in crops (Saxena et al., 2020). Furthermore, a LEfSe analysis focused on the bacterial OTUs common to both root chambers of the two treatments. It revealed a significant increase in Burkholderiales in the continuous cropping method and Chloroplast in the crop rotation approach. As a leading and plant pathogenic microbe, Burkholderiales is prevalent in numerous continuous cropping soils (Fan et al., 2022; Qiao et al., 2017). The growing prevalence and comparative enhancement of Burkholderiales play a significant role in the continuous challenges encountered in cropping. Chloroplast cells possess nitrogen fixation capabilities and can increase soil fertility (Touliabah et al., 2022).

### 4.2 Soybean soil metabolites

Technologies in non-specific metabolomics can label a vast array of metabolites in soil specimens, accumulate numerous metabolites, and conduct exact metabolite examinations in subsequent phases (Turner et al., 2013; Rivas-Ubach et al., 2013; White et al., 2017). The interplay among microbial metabolic products, root secretions, and substances from certain living beings further influences soil’s internal environmental recycling (Raaijmakers et al., 2010). A planting system can transform the microbial composition of both the crop soil and the soil metabolites (Ku et al., 2022; Ma et al., 2016; Hoeffner et al., 2021). The research demonstrated that sustained cropping modified levels of organic acids, sugars, organic compounds, lipids, unsaturated fatty acids, alcohols, and various other compounds in soil, resulting in alterations in metabolic pathways including the transformation of pentose and glucuronic acid in the soil, as well as microbial and steroid biosynthesis, synthesis of secondary metabolites, and the processing of starch and sucrose.

The roots of plants emit diverse substances, such as organic fatty acids, and bacteria are also capable of breaking down organic acids during their biological processes (Peña, 2022; Li et al., 2012). After persistent cropping, there is an increase in the soil’s organic fatty acids, leading to further deterioration of the soil’s conditions and resulting in diseases transmitted by the soil (Wang et al., 2022). The research found that the levels of eicosanoid acid and pentadecanoic acid in continuous cropping rotation were significantly higher than in crop rotation. An increase in these two types of fatty acids can lead to continuous challenges in cultivating soybeans. A significant amount of soil metabolites are carbohydrates, acting as direct carbon sources, which supply the energy necessary for soil microorganisms to conduct their essential functions (Behera and Wagner, 1974; Xingdong et al., 2023). During both the continuous cropping and crop rotation practices, sugar levels were higher than in the continuous cropping approach, potentially contributing to the increased presence of soil bacteria in each method. The research found that continuous cropping enhanced glycerol levels compared to regular crop rotation. Plants can release glycerol or it can be processed by microorganisms, which serves to obstruct cell membrane penetration and supply energy to soil microbes (Bolen, 2001; Shen et al., 1999; Nikel et al., 2015). Conversely, research involving Arabidopsis rhizosphere soil indicates an elevation in glycerol levels can impede the regular growth and development of Arabidopsis roots (Hu et al., 2014).

### 4.3 Soybean soil factors bacterial interaction relationship

Earlier research indicates a close correlation between soil environmental elements and the comparative prevalence of soil microbes (Xiong and Lu, 2022). This research found that during crop rotation, elements such as urease, leucine aminopeptidase, N-acetylglucosamine, available phosphorus, organic matter, organic carbon, and total potassium markedly alter the prevalence of Rhodobacter and Gemmatimonas genera. Additionally, in sustained crop, the presence of potassium, sucrase, and catalase markedly influences Bradyrhizobium, a type of slow-growing rhizobia.

### 4.4 Correlation between soil bacteria and soil metabolites

Swenson noted a notable interconnection and interaction between the makeup and concentration of soil metabolites and the makeup and variety of soil bacterial ecosystems (Swenson et al., 2015). The research revealed substantial growth of Cyanobacteria in crop rotation, in contrast to Burkholderiales, which demonstrated considerable enhancement in continuous cropping (Figures 2–9). Cyanobacteria, a vital microbial entity on Earth, possess a high metabolic capability. This element plays a crucial role in the Earth’s surface soil and is extensively involved in the nitrogen fixation process of photosynthesis. Cyanobacteria is a bacterium that photosynthesizes nitrogen (Cano-Díaz et al., 2021). A direct link exists between Cyanobacteria and Benzyl Alcohol, known to fight bacteria in the soil by interfering with the movement of bacterial cell membranes (Yano et al., 2016); conversely, Cyanobacteria have an inverse relationship with Sorbitol, and a rise in Sorbitol levels impedes photosynthesis (Gao et al., 2014). Burkholderiales, frequently encountered in continuous cropping diseases, potentially coexist with Fusarium and lead to soil illnesses (Stopnisek et al., 2016). This research shows a direct correlation between Burkholderiales and D-arabinitol, D-pinitol, pentadecanoic acid, propanedionic acid, and fatty acids such as pentadecanoic acid and propanedionic acid (Smedman et al., 1999). Burkholderiales serve as substrates for sugar and fatty acid metabolic processes and play a role in breaking down D-arabitol and D-pinitol (Alvarez-Santullano et al., 2021).

## 5 Conclusion

Exploring the link between soil bacteria and metabolites involved applying 16S rDNA sequencing and soil metabolomics methods to assess alterations in microbial composition and metabolomics across various root areas during continuous soybean cropping and corn rotation. An analysis of the varying abundance reveals that continuous agricultural practices diminish the diversity and relative prevalence of soil bacteria. Moreover, the continuous cropping of soybeans and the practice of corn–soybeans rotation have both modified the makeup of the soil’s bacterial populations. Soil microorganisms within the rhizosphere exhibit a diminishing concentration of bacteria from its farthest point to the endosphere. Findings from metabolomics revealed that both continuous cropping and rotating soybeans in corn altered the kinds of soil metabolites, disrupted the metabolic rates of the soil, and decreased sucrose levels. The variation in these metabolites demonstrated a substantial correlation with shifts in bacterial populations, including Burkholderiales. This study’s findings validate a tight linkage between the composition of soil bacteria and its metabolites, offering a theoretical framework to ease continuous soybean cropping challenges, sensible corn–soybean rotation, and enhancing future soybean yields.

## Data Availability

The metabarcoding data sets of fungal 16S amplicons are accessible under NCBI BioProject PRJNA1209829.
